# Smartphone Addiction, Screen Time, and Physical Activity of Different Academic Majors and Study Levels in University Students

**DOI:** 10.3390/ijerph22020237

**Published:** 2025-02-07

**Authors:** Wannisa Kumban, Salila Cetthakrikul, Anoma Santiworakul

**Affiliations:** 1 Department of Physical Therapy, School of Allied Health Science, Walailak University, 222, Thasala, Thai-buri, Nakhon Si Thammarat 80160, Thailand; wannisa.ku@wu.ac.th (W.K.); salila.ce@wu.ac.th (S.C.); 2 Movement Science and Exercise Research Center, Walailak University (MoveSE-WU), 222, Thasala, Thai-buri, Nakhon Si Thammarat 80160, Thailand

**Keywords:** smartphone addiction, screen time, physical activity, academic major, study level, university student

## Abstract

Smartphone addiction is increasing among university students. This study aims to explore the differences in screen time and physical activity among university students with and without smartphone addiction, considering their study majors and levels. One hundred and twenty participants from sixteen faculties were included, divided into three academic majors (health sciences, social sciences and humanities, and science and technology). Data were collected using a screen time behavior questionnaire and a screen time application. Physical activity was measured using the Global Physical Activity Questionnaire (GPAQ). This study demonstrated that the smartphone addiction group had higher screen time on both smartphones (*p* = 0.02) and other devices (*p* = 0.04). Students in the social sciences and humanities majors had insufficient physical activity according to WHO recommendations. The results showed no correlation between screen time and physical activity, study time, academic major, or study level (*p* ≥ 0.05). However, a low correlation was observed between physical activity and study time (r^2^ = 0.27; *p* < 0.05), as well as study level (r^2^ = −0.31; *p* < 0.05). Our findings show that the total screen time for university students with and without smartphone addiction exceeded 8 h per day. Real-life activities should be promoted to reduce screen time. Encouraging physical activity among senior students and those in social science and humanities majors is crucial for fostering healthy behaviors in the future. Physical education and recreational activities should be emphasized in these groups, along with the development of programs or class activities designed to promote physical activity and reduce screen time.

## 1. Introduction

Screen time on smartphones, tablets, computers, and other devices has become an essential part of daily life in today’s digital world. Smartphone ownership has increased to about half or more of the world’s population over the past decade [1]. Smartphone usage is exponentially increasing among university students worldwide. Young adults aged from 18 to 24 have the highest smartphone ownership rates and spend more time on smartphones [2]. Studies conducted among university students in many countries have found that smartphone ownership among these students is very high. The increased use of screen technology has been suggested as a contributing factor to this trend. A survey by Thailand’s National Statistical Office in 2023 found that 99.2 percent of youth aged 18–24 own a mobile phone, 99.9 percent of which are smartphones, and 99 percent use them to access the internet [3]. Mora-Monteros et al. surveyed screen usage among youths in Switzerland between 2012 and 2020. The results showed that smartphone usage increased, with 71.7% of youths using smartphones in 2020 compared with 23.2% in 2012. Additionally, young people’s screen time dramatically increased, with adolescents spending more than 4 h on screens in 2020 compared with 2012 [4]. Thai youth are also increasingly more likely to use social media and smartphones and tend to become addicted to the internet and social media [5]. Young people are particularly active users of screen-based devices, engaging in activities such as smartphone use, tablet usage, notebook browsing, video gaming, and watching movies and TV series [6]. These individuals utilize their devices to browse the internet, watch videos, check updates on social networking sites, and learn both in and out of the classroom [7].

The availability of smart devices and the development of technology increase the amount of time spent sitting. All forms of recreational screen time can potentially contribute to sedentary behavior. It is crucial to understand the health implications of social media and electronic device use given their prevalent use and their purported links to reduced mental health, impaired sleep (both negatively associated with sleep quantity and quality), musculoskeletal symptoms, urinary incontinence in females, and risk of motor vehicle accidents [8]. Excessive sedentary behavior has a strong correlation with chronic diseases such as cardiovascular diseases, diabetes, obesity, and mental health problems. The indirect impact of excessive sedentary behavior on mental health problems is mediated by lifestyle choices and social interactions [9].

A survey comparing the physical activity and sedentary behavior of the Thai population in 2009 and 2015 found that Thai people of all ages, including the 18–24 age group, experienced a decrease in physical activity and an increase in sedentary behavior [10]. Moreover, greater screen time use per day is inversely related to the number of days per week university students engage in physical activity. High smartphone use among college students may act as a barrier to physical activity [11]. Many factors influence screen time and physical activity in university students, including lifestyle behaviors and daily screen usage. Previous research has shown that factors related to sedentary behavior and physical activity, both during and outside classes, vary between different academic subgroups of university students [12,13,14,15,16,17]. A study by Dayi et al. observed the highest incidence of physical activity among students in the School of Sports Sciences and Technology (74.5%). Conversely, the highest levels of physical inactivity were observed among students in the Faculty of Nursing [15]. A study by El Gilany et al. found that physical inactivity levels were 1.8 times higher in students studying in social science departments than in medical school students [16]. A multiple logistic regression analysis by Q-En Chung et al. indicated that the program of study was not related to physical activity levels among medical and health sciences students in Malaysia [18]. Edelmann et al. studied physical activity and sedentary behavior across different fields of study and academic years. They found a significant difference between students enrolled in natural sciences, mathematics, and informatics; those in social sciences, media, and sports; those in medicine; and those in education. Regarding sedentary behavior, they reported that students in natural sciences, mathematics, and informatics had the highest average sitting time of 7 h and 47 min, while students in social sciences, media, and sports had the lowest average sitting time of 7 h and 8 min. Additionally, first-year students reported significantly lower levels of physical activity than students in subsequent years. However, sedentary behavior did not differ significantly between academic years [17]. Zhou et al. found that freshman, sophomore, and junior sports university students showed no differences, spending almost half of their sedentary time studying, including attending classes and working on homework [13]. An increase in the year of study decreased physical inactivity, while an increase in social status increased physical inactivity [16]. In a previous study, lifestyle differences between study majors and year levels were associated with differences in physical activity levels. Study major, enrollment status, and year of enrollment may be relevant factors that could explain variations in sedentary behavior levels [14]. Previous studies have generally observed sedentary behavior and physical activity in relation to academic factors among university students but have not directly measured related screen time behavior. Recently, screen time usage has been increasing worldwide, including among university students. However, few studies have explored the relationship between screen time, physical activity, and academic factors in university students. To examine the impact of screen time on physical activity in relation to academic factors, this study aims to explore the influence of study major, study time, and year level on screen time and physical activity among university students.

## 2. Materials and Methods

### 2.1. Study Design and Setting

This research uses a cross-sectional study design, recruiting both male and female 18–25-year-old Walailak University students from all study years. The inclusion criteria for the study were as follows: the subject was interested in participating in the study and used a smartphone/tablet with an Android or iOS system; there is a screen time application available in the settings menu for both Android and iOS. The exclusion criteria were as follows: participants with diseases of the musculoskeletal, nervous, or cardiopulmonary system that affect physical activity; participants taking drugs that cause drowsiness, such as sedatives and antihistamines, as these may affect physical activity; and participants who suffered injuries during data collection or were unable to collect data for 7 days.

Data collection took place between January and April 2022. An announcement was made to invite and encourage students to participate through online media channels, such as Facebook and Instagram, and notices were posted on public relations boards at various points in the university, including academic buildings, cafeterias, and student dormitories. All questionnaires were individually completed by the participants in the presence of a researcher online via the Zoom application. This study protocol was approved by the Ethics Committee in Human Research, Walailak University (WUEC-22-007-01). Participants were informed of the details of the study and its data security protocols, and consent was obtained before the questionnaires were filled out.

### 2.2. Sample Size and Sampling

Using the G*power program, the sample size was calculated from the entire university population, which consists of 16 faculties divided into 3 academic majors (health sciences, social sciences and humanities, and science and technology), according to the study by Towne et al. [19]. Cluster random sampling was used to recruit students from each academic major across all four years (1st to 4th year) in health sciences, social sciences and humanities, and science and technology. Participants were then divided into two groups (screen addiction and non-screen addiction behaviors) based on the Thai version of the Modified Smartphone Addiction Scale—Short Version (SAS-SV). There were 43 men (35.8%) and 77 women (64.2%). This study recruited junior students (35 students (29.2%) from the first academic year and 32 students (26.7%) from the second academic year) and senior students (27 students (22.5%) from the third academic year and 26 students (21.7%) from the fourth academic year). In total, 45 students (37.5%), the majority, were health sciences students; 37 (30.8%) were science and technology students; and 38 (31.7%) were social sciences and humanities students.

### 2.3. Instruments and Procedures

During the research data collection process, we considered potential biases that could affect data quality. To address recall bias and social desirability bias, where participants may forget, overestimate, or underestimate their behaviors, particularly physical activity measured by the GPAQ, the data collector informed participants in advance about the need to record their physical activities for seven days. This was achieved by clearly explaining the required information for the GPAQ beforehand, allowing participants to prepare accurate responses. To mitigate response bias, the study employed face-to-face communication via the Zoom application to facilitate clear interactions between the data collector and participants. However, anonymous surveys were used to ensure that participants’ data remained confidential and unknown to the data collector. Additionally, standardized survey instructions were communicated by the data collector. For data quality assurance, the researcher provided detailed instructions throughout the questionnaire collection process, guiding participants on how to complete the questionnaire and allowing them to ask questions until they fully understood. Participants were also encouraged to seek clarification at any point if needed.

To collect the characteristics of the participants, a questionnaire was used to gather demographic information, including age, gender, BMI, academic major, academic year, study time, underlying health conditions, and medications taken. Then, the Thai version of the Modified Smartphone Addiction Scale—Short Version (SAS-SV) [20] questionnaire was used to assess smartphone addiction risk [21,22], classifying screen addiction behaviors. The reliability of the TH-SAS-SV with internal consistency was calculated as 0.94 for the total scale using Cronbach’s alpha, ranging from 0.76 to 0.97 for the subscales [20,23]. The SAS-SV comprises 10 questions, each employing a Likert-type rating of 1–6 (1 = strongly disagree; 6 = strongly agree). The cutoff score is 31 points for men and 33 points for women, classifying participants as addicted or high-risk smartphone users [22].

In 7 days of recording, the screen time behavior questionnaire was used to collect data regarding the amount of time spent per day using a TV, tablet, smartphone, desktop computer, laptop, etc. Moreover, screen time was assessed using the screen time application included on smartphones and tablets with Android and iOS systems to record usage per day. Each morning on the data collection days, participants were instructed to meet with the data collectors via Zoom at specified times to submit the screen time behavior questionnaire. They were also required to take screenshots from their mobile phones and tablet applications and send them to the research data collector to ensure no data were forgotten or missed.

At the end of the 7-day screen time data collection period, Physical activity was measured using the Global Physical Activity Questionnaire (GPAQ) via Zoom. The GPAQ was developed in 2002 and validated by the World Health Organization (WHO) to systematically monitor global physical activity levels; it is widely used and is accurate in measuring physical activity [24,25]. The GPAQ is a self-reported questionnaire comprising 16 questions and covers several components of physical activity, such as intensity, duration, and frequency in the past 7 days. There are 3 domains, including activity at work, travel to and from places, and recreational activities. The validity and test–retest reliability levels of the Thai version of GPAQ are acceptable (r = 0.77) [26]. This study presents the GPAQ data in minutes per week.

### 2.4. Statistical Analysis

The SPSS version 20 statistics program was used for statistical analysis. The defining characteristics of the demographic information were number, percentage, means, and standard deviation (SD). The Kolmogorov–Smirnov test was used to verify the normality of the quantitative variables. The non-parametric Mann–Whitney U test was used to evaluate the differences between the smartphone addiction and non-smartphone addiction groups. The Kruskal–Wallis H test was used to determine if there were statistically significant differences between academic majors. The Mann–Whitney U and Chi-square tests were used to evaluate differences in physical activity and screen time between senior and junior students. Spearman’s rank correlation test was used to evaluate the relationship between variables. *p*-Values less than 0.05 were considered statistically significant.

## 3. Results

This study aims to explore the differences in screen time and physical activity between university students with and without smartphone addictions, considering their study majors and levels. Smartphone addiction was assessed to divide participants into smartphone addiction and non-addiction groups. Physical activity in terms of MVPA, screen time (including smartphone usage, other device usage, and total screen time), and academic parameters (including study time, study major, and study level) were compared, and correlations were analyzed.

The normality test showed that all the following variables had non-normal distributions: age, academic year, body mass index (BMI), study time (hours per week), GPAQ (minutes per week), smartphone screen time (minutes per day), screen time on other devices (minutes per day), and total screen time (minutes per day). Thus, the Mann–Whitney U test was used to evaluate the differences between smartphone addiction and non-smartphone addiction. The Kruskal–Wallis H test was used to determine whether there were statistically significant differences between academic majors.

**Figure 1:**
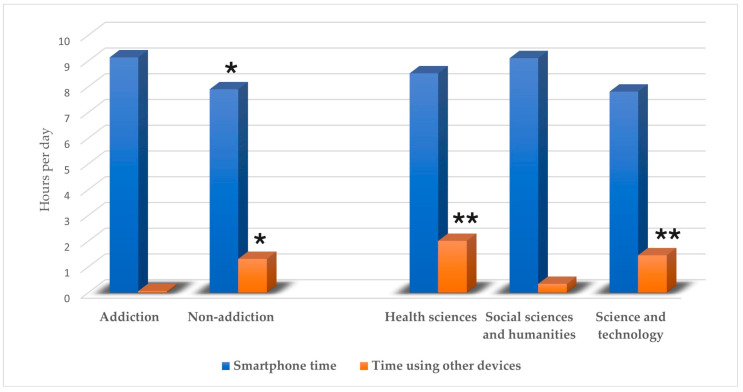
Screen time per day among the smartphone addiction group and academic major groups. * Significant difference from the addiction group, *p*-value < 0.05; ** Significant difference from social sciences and humanities majors, *p*-value < 0.05.

One hundred and twenty students participated in this study. In total, 57 students (47.5%) had screen addiction, and 63 (52.5%) did not. The percentages of smartphone addiction in the health sciences, social sciences and humanities, and science and technology majors were 46.67%, 50%, and 45.94%, respectively. The descriptive data are shown in Table 1. All participants used smartphones, and eight used other devices, such as tablets, computers, or laptops. The reported daily smartphone screen time ranged from 0.70 to 22.98 h, while the usage duration for other devices ranged from 0.33 to 1.30 h per day. The total screen time across all devices ranged from 1.67 to 18.02 h per day. Study time (hours per week) and screen time using other devices (hours per day) showed significant differences between the three academic majors. Comparing each major, it was shown that science and technology majors were associated with a significantly higher study time than health sciences majors (*p* = 0.003) and social sciences majors (*p* = 0.001). The Mann–Whitney U test revealed that smartphone usage (hours per day), time spent using other devices (hours per day), and total screen time (hours per day) were significantly higher in the smartphone addiction group compared to the non-smartphone addiction group (*p* = 0.02, *p* = 0.04, and *p* = 0.04, respectively). Additionally, students majoring in health sciences and science and technology had higher daily screen times for using other devices than students majoring in social sciences and humanities (*p* = 0.005, *p* = 0.037, respectively), as shown in Figure 1.

Table 2 compares physical activity, screen time, and smartphone addiction between senior and junior students. The Mann–Whitney U test revealed junior students had significantly more study time and physical activity than senior students (*p* > 0.001), but senior students showed significantly more tablet or notebook screen time than junior students (*p* > 0.01). The number of students with smartphone addiction between junior and senior university students was not significantly different (*p* ≥ 0.50).

The correlation results showed no relationship between screen time and study time, academic major, study level, or physical activity among the participants (*p* ≥ 0.50). There was no correlation between physical activity and academic major. However, there was a low positive correlation (r^2^ = 0.27; *p* < 0.05) between MVPA (minutes per week) and study time (*p* = 0.03) and a low negative correlation (r^2^ = −0.31; *p* < 0.05) between study level and MVPA (minutes per week) (*p* = 0.001) (Table 3).

## 4. Discussion

This study aimed to explore the differences in screen time and physical activity between university students with and without smartphone addiction, considering their study majors (health sciences, social sciences and humanities, and science and technology) and study levels (junior and senior). One hundred and twenty students participated in this study. In total, 57 students (47.5%) had screen addiction, and 63 (52.5%) did not.

Our findings showed that there was no statistically significant difference in age, BMI, study time, or physical activity between students with and without screen addiction. The smartphone addiction and non-smartphone addiction groups had BMI values that were not statistically different. Both groups had MVPA (moderate-to-vigorous physical activity) values that were not significantly different and exceeded 150 min per week, meeting the WHO recommendation and classifying them as active [27]. However, both groups reported screen times of more than 8 h per day, with similar study times of 25.57 and 26.15 h per week. Considering the combination of high physical activity and prolonged screen use in both the smartphone addiction and non-smartphone addiction groups, the sample was classified as active + sedentary. Previous research has shown a relationship between physical activity and BMI in university students [28]. However, in this active + sedentary group, no significant relationship was found between physical activity levels and overweight/obesity [29]. Among university students in early adulthood in the screen addiction group, the average age was 20.70 years; the average age for those without screen addiction was 20.21 years. Previous studies have found that smartphone addiction is more prevalent among adolescents aged 15–16 years [30]. Nowadays, communication and technology play a crucial role in society. As a result, smartphone usage has increased among university students and young adults. Previous studies have found that smartphone use varies depending on individual purposes, with most usage focused on social media, internet searches, messaging, and similar activities [31]. The present study found a significant difference in daily smartphone usage times between participants with and without smartphone addiction. Additionally, we found that 47.5% of participants exhibited screen addiction, spending more than 8 h per day using smartphones and other devices. This is consistent with previous studies showing that 61% of university students have high smartphone usage, spending over 8 h per day on this activity [32]. Another study reported that 49% of 688 Lebanese university students exhibited excessive smartphone use (≥5 h per weekday) [33]. The threshold for excessive smartphone usage, indicating addiction, has been set at 4.62 h per day [34]. A study by Kim et al. also reported a relationship between smartphone usage hours and smartphone addiction [35].

We found that physical activity levels, as measured by the GPAQ, did not differ between participants with and without screen addiction. We also showed that participants in both groups had an average MVPA that met the adequate physical activity level (at least 150 min per week). According to the World Health Organization (WHO), adults aged 18–64 years are recommended to engage in at least 150 min of moderate physical activity per week (or at least 75 min of vigorous physical activity, or an equivalent combination of both) [27]. This is consistent with previous studies that also reported no difference in physical activity levels between individuals with and without smartphone addiction [36,37]. By contrast, some studies have found that individuals with smartphone addiction or those at high risk of it tend to be less physically active, as smartphone addiction can disrupt physical activity routines [30,35,38]. As such, high screen time alone may not provide sufficient variability to cause negative effects or lower physical activity levels. In this regard, differences in usage amounts and frequencies observed in various studies may contribute to these results.

In terms of academic majors, students in the social sciences and humanities branch had insufficient physical activity levels, as measured by the GPAQ (MVPA < 150 min per week), compared with those in health sciences and science and technology majors. Factors influencing physical activity levels among university students include university-specific characteristics, and psychological, emotional, cognitive, environmental, and sociocultural factors [39]. Several psychological factors, such as perceived enjoyment, self-discipline, values, norms, beliefs, and time management, have been found to influence both physical activity and sedentary behavior. Certain sedentary activities, such as spending time on social media via smartphones, tablets, or computers, have been identified as compulsory sedentary behaviors and barriers to physical activity among university students [39]. The current study provides useful insights into how different levels of physical activity knowledge are associated with physical activity behavior. The findings demonstrate an association between physical activity and knowledge of diseases related to inactivity. Participants who overestimated the increased risk of disease due to inactivity were more active than those who underestimated it. It is recommended that physical activity interventions and health promotion initiatives improve the awareness of types of diseases associated with inactivity [40]. A study by Ayfer Dayi et al. found that the majority of students understood the benefits of performing physical activities, with students in the Faculty of Medicine having more knowledge concerning the advantages of physical activity than other students [15]. The health sciences group had a significantly higher prevalence of intense physical activity (IPAQ > 1500 METS-min/week) than the education-related degree and arts and management professional groups. On the other hand, no significant differences were observed regarding the prevalence of sedentary lifestyles (IPAQ < 600 METS-min/week) between health sciences, education-related degrees, and arts and management professionals [41]. This study found that junior university students had significantly higher levels of physical activity than senior university students. This aligns with studies by Romero-Blanco et al. [42] and Sevil et al. [43], which demonstrated that students in higher years of study are less active. Senior students also spend less time engaging in physical activity and more time using other devices, likely due to a heavier study workload [14]. Another reason could be that first-year students might have practiced sports in high school and kept up the habit [42]. In Egypt, a study indicated that physical activity decreased in later years of study, whereas increased social status led to higher physical inactivity [18]. 

The results showed no correlation between screen time and physical activity. Screen time included using a smartphone, a notebook, or a tablet throughout the day. This result was similar to that of a previous study [44] showing no relationship between daily physical activity and overall screen time among young adolescents. However, our study could not determine a cause-and-effect relationship between physical activity and screen time due to its cross-sectional design. In contrast, a multivariable logistic analysis in 2018 showed that being physically active was negatively associated with high screen times [45]. Additionally, the evidence was unclear on whether screen time directly affects physical activity. However, the longer the screen time, the shorter the sleeping time. Sedentary behavior also increases in children and adolescents with poor sleeping habits [46]. Study time, academic major, and study level had no association with overall screen time in this study. The factors influencing smartphone use among university students are mood, available time, activities, and whether they are alone or with others [47]. There was a low positive correlation between physical activity and study time and a low negative correlation between physical activity and study level. This finding might be attributable to junior students in this study exhibiting higher physical activity levels than senior students. A qualitative study conducted in 2015 using focus group discussions revealed that factors influencing physical activity behavior in university students included time schedules (study time and examination periods) and their level of self-discipline [39]. This study differed from previous studies that showed that higher years of study tend to be accompanied with a decrease in physical inactivity, whereas an increase in social status leads to higher physical inactivity [18]. Moreover, research on interpersonal factors, focusing on university students, notes that social support from friends influences physical activity [39]. All participants stayed on campus, so students may experience less parental modeling but more peer modeling. However, there was no relationship between physical activity levels and academic majors. This might be because academic major was not a primary factor influencing physical activity levels [39].

This study aimed to determine the differences and relationships between smartphone addiction, screen time, physical activity, academic major, and study level among university students. However, there were four limitations. First, there was a lack of demographic data, including socioeconomic status and detailed information about activities during and outside of class, which might affect the physical activity levels and screen time of university students. To fully address the factors related to physical activity, future studies should collect this information. Second, data on the types of screen time use should be included to present different screen time modes. Such results could provide valuable data for promoting or preventing the overuse of screen devices. Third, a self-report questionnaire was used, which is a subjective measure and might introduce biases, such as recall bias when recalling information retrospectively, overestimation when providing responses, or misunderstandings while filling out the questionnaire. Although the researcher controlled for potential biases during the collecting process, for future studies, we recommend adding objective measures to reduce biases. The final limitation was that the data were collected from only one university, which may limit the generalizability of our findings to universities in different environments. Therefore, multiple universities should be included in future studies.

## 5. Conclusions

This study demonstrated that university students with smartphone addiction had high screen times, using both smartphones and other devices more frequently. However, the total screen time for university students with and without smartphone addiction exceeded 8 h per day. No differences in physical activity were observed between the addiction and non-addiction groups, as measured via the GPAQ. Students in social sciences and humanities majors had insufficient physical activity (MVPA < 150 min/week) compared with those in health sciences and science and technology majors. However, there were no differences in smartphone usage or total screen time between these groups. Junior students were more physically active, but there were no differences in smartphone usage or total screen time. Additionally, correlations between physical activity, study time, and study level were observed. Considering the limitations of this study, future research should address them by incorporating objective measures, expanding to multiple universities, and considering additional demographic factors. Promoting physical activity among senior students and those in social sciences and humanities majors will be crucial for fostering healthy behaviors in the future. Real-life activities should be encouraged to reduce screen time, and physical education and recreational activities should be emphasized in these groups. Additionally, programs or class activities designed to promote physical activity and reduce screen time should be developed.

## Figures and Tables

**Table 1:** Comparison of participants’ characteristics, physical activity, and screen time.

Descriptive	Total (n = 120)	Smartphone Addiction		*p*-Value ^a^	Academic Major			*p*-Value ^b^
	Mean (SD)	Yes (n = 57)	No (n = 63)		Health Sciences (n = 45)	Social Sciences and Humanities (n = 38)	Science and Technology (n = 37)	
Age (years)	20.44 (1.31)	20.70 (1.27)	20.21 (1.32)	0.05	20.57 (1.21)	20.57 (1.28)	20.13 (1.43)	0.17
Body mass index (kg/m^2^)	22.91 (4.84)	23.61 (4.97)	22.29 (4.66)	0.09	22.47 (4.59)	22.15 (4.88)	24.24 (4.95)	0.10
Study time (hours per week)	25.88 (8.63)	25.57 (9.37)	26.15 (7.96)	0.41	24.57 (4.97)	23.78 (8.64)	29.62 ^#,$^ (10.87)	0.001 *
MVPA (minutes per week)	204.65 (240.69)	213.59 (243.30)	196.57 (239.97)	0.77	241.55 (267.40)	136.36 (139.17)	229.91 (277.35)	0.45
Smartphone time (hours per day)	8.49 (3.33)	9.14 (2.62)	7.90 (3.79)	0.02 *	8.52 (3.17)	9.11 (3.49)	7.81 (3.31)	0.18
Time using other devices (hours per day)	0.68 (2.14)	0.06 (0.49)	1.31 (2.99)	0.04 *	2.01 ^#^ (3.58)	0.34 (1.45)	1.45 ^#^ (3.18)	0.005 *
Total screen time (hours per day)	9.16 (3.03)	9.75 (2.50)	8.62 (3.38)	0.04 *	9.22 (3.39)	8.98 (2.18)	9.26 (3.38)	0.90

* *p*-value < 0.05; ^#^ significant difference from social sciences and humanities majors; ^$^ significant difference from health sciences, ^a^ Mann–Whitney U test; ^b^ Kruskal–Wallis H test.

**Table 2:** Comparison of physical activity and screen time between senior and junior students.

Descriptive	Level		*p*-Value ^a^
	Junior (n = 67)	Senior (n = 53)	
Study time (hours per week)	28.08 (5.98)	23.09 (10.53)	<0.001 *^,a^
MVPA (minutes per week)	267.80 (258.89)	124.83 (189.41)	<0.001 *^,a^
Smartphone time (hours per day)	8.69 (3.41)	8.23 (3.24)	0.69 ^a^
Time using other devices (hours per day)	0.06 (0.49)	0.80 (2.31)	0.01 *^,a^
Total screen time (hours per day)	8.93 (3.23)	9.44 (2.77)	0.44 ^a^
Smartphone addiction (Yes)	30 (44.77%)	27 (50.94%)	0.50 ^b^

* *p*-value < 0.05; ^a^ Mann–Whitney U test; ^b^ Chi-square test.

**Table 3:** Correlations between physical activity and study time, academic major, and study level.

	Correlation	Study Time	Academic Major	Study Level
MVPA (minutes per week)	r^2^	0.27 *	−0.04	−0.31 *

* *p*-value < 0.05, Spearman’s sign rank test.

## Data Availability

The data are available upon request from the authors.
